# Improved Visualization of Focal Cortical Dysplasia With Surface-Based Multiparametric Quantitative MRI

**DOI:** 10.3389/fnins.2020.00622

**Published:** 2020-06-16

**Authors:** Michelle Maiworm, Ulrike Nöth, Elke Hattingen, Helmuth Steinmetz, Susanne Knake, Felix Rosenow, Ralf Deichmann, Marlies Wagner, René-Maxime Gracien

**Affiliations:** ^1^Department of Neurology, Goethe University, Frankfurt, Germany; ^2^Department of Neuroradiology, Goethe University, Frankfurt, Germany; ^3^Brain Imaging Center, Goethe University, Frankfurt, Germany; ^4^Center for Personalized Translational Epilepsy Research Consortium (CePTER), Frankfurt, Germany; ^5^Department of Neurology, Philipps University of Marburg, Marburg, Germany; ^6^Epilepsy Center Frankfurt Rhine-Main, Center of Neurology and Neurosurgery, Goethe University, Frankfurt, Germany

**Keywords:** epilepsy, focal cortical dysplasia, quantitative magnetic resonance imaging, neuroimaging, brain imaging

## Abstract

**Purpose:**

In the clinical routine, detection of focal cortical dysplasia (FCD) by visual inspection is challenging. Still, information about the presence and location of FCD is highly relevant for prognostication and treatment decisions. Therefore, this study aimed to develop, describe and test a method for the calculation of synthetic anatomies using multiparametric quantitative MRI (qMRI) data and surface-based analysis, which allows for an improved visualization of FCD.

**Materials and Methods:**

Quantitative T1-, T2- and PD-maps and conventional clinical datasets of patients with FCD and epilepsy were acquired. Tissue segmentation and delineation of the border between white matter and cortex was performed. In order to detect blurring at this border, a surface-based calculation of the standard deviation of each quantitative parameter (T1, T2, and PD) was performed across the cortex and the neighboring white matter for each cortical vertex. The resulting standard deviations combined with measures of the cortical thickness were used to enhance the signal of conventional FLAIR-datasets. The resulting synthetically enhanced FLAIR-anatomies were compared with conventional MRI-data utilizing regions of interest based analysis techniques.

**Results:**

The synthetically enhanced FLAIR-anatomies showed higher signal levels than conventional FLAIR-data at the FCD sites (*p* = 0.005). In addition, the enhanced FLAIR-anatomies exhibited higher signal levels at the FCD sites than in the corresponding contralateral regions (*p* = 0.005). However, false positive findings occurred, so careful comparison with conventional datasets is mandatory.

**Conclusion:**

Synthetically enhanced FLAIR-anatomies resulting from surface-based multiparametric qMRI-analyses have the potential to improve the visualization of FCD and, accordingly, the treatment of the respective patients.

## Introduction

Focal cortical dysplasia (FCD) is a developmental cortical malformation with a high epileptogenic potential, often causing drug-refractory epilepsy (Gaitanis and Donahue, 2013). Accordingly, surgical treatment is often required for these patients. Diagnostics and pre-surgical evaluation include MRI to identify the FCD and estimate its extent and localization. This information is crucial as the complete resection of the lesion is an important factor for postsurgical outcome (Fauser et al., 2004; Lerner et al., 2009; Kabat and Król, 2012).

Typical MRI features of FCD include a blurring of the junction between cortical gray matter (GM) and white matter (WM), a thickening of the cortical layer, hyperintensities in subcortical WM in T2-weighted datasets and abnormal patterns of gyri and sulci (Colombo et al., 2003; Kabat and Król, 2012). However, MR-changes are often subtle and detection and evaluation of the location and extent of the lesions can be challenging with conventional MRI-techniques (Colombo et al., 2003; Kabat and Król, 2012; Hong et al., 2016). Still, clinicians need this information for treatment decisions.

Thus, post-processing techniques for conventional MRI-data have been developed to optimize the visualization of FCD. Methods described by Kassubek et al. (2002) and Huppertz et al. (2005) analyze the junction between GM and WM and the extension of the GM by normalizing T1-weighted datasets of patients with epilepsy to allow for a comparison with a control cohort. Furthermore, machine learning was used in a previous study for automated FCD detection, analyzing conventional MRI parameters (Hong et al., 2014).

Approaches using conventional MRI-data usually require steps for intensity-standardization. In contrast to conventional MRI-techniques, quantitative MRI (qMRI) measures tissue-parameters such as the T1- and T2-relaxation times or the proton density (PD) free from hardware-effects (Cercignani et al., 2018) and the resulting inhomogeneities. Accordingly, qMRI could serve as a promising basis for FCD-visualization. Nöth et al. (2020) developed a method for FCD-detection based solely on quantitative T1-data and on voxel-wise analysis of the whole brain.

In the present preliminary technical study, it was aimed to develop a method which allows for an improved visualization of FCD, using a multimodal qMRI-approach. The potential advantage is that the technique integrates information from different complementary parameters (T1, T2, and PD). The method utilizes a reconstruction of WM and pial surfaces and boundary-based analysis techniques which integrate information about the course and orientation of the WM and pial surfaces when reading parameter values and measuring the cortical thickness. The results of the calculation are used to highlight FCD areas in FLAIR datasets. As a consequence, this method has the potential to aid visual assessment of image data, thus helping to reduce the number of undetected lesions, potentially allowing for a more effective treatment.

In summary, the purpose of this study was to develop and describe the method, to show representative data and to quantify the improvement in image contrast via comparison with conventional MRI datasets, using a regions of interest (ROI) based analysis.

## Materials and Methods

### Participants

MRI-acquisition was performed for 10 patients with neuroradiologically diagnosed FCD based on clinical MRI-data (three females, age: range 18–55 years, mean ± SD: 29.6 ± 11.7 years) and five healthy subjects (three females, age: range 19–34 years, mean ± SD: 24.4 ± 5.1 years). The studies involving human participants were reviewed and approved by the respective local board (Ethik-Kommission des Fachbereichs Medizin des Universitätsklinikums der Goethe-Universität). The patients/participants provided written informed consent to participate in this study. The study was performed according to the principles formulated in the Declaration of Helsinki.

The investigated cohort and the data obtained overlap with a previous study presenting a different method for FCD-detection based solely on T1-maps and on a voxel-wise analysis across the whole brain (Nöth et al., 2020). In the current study, a completely different approach is described which utilizes multiparametric qMRI data and a surface-based analysis. Furthermore, some data obtained on subjects with FCD overlap with previous studies with different aims evaluating improved synthetic T1-weighted datasets for tissue-segmentation (Gracien et al., 2019) and assessing normal-appearing cortical tissue in patients with FCD via T2-relaxometry (Ahmad et al., 2020).

### Data Acquisition and qMRI-Mapping

A 3 Tesla (T) MRI-scanner “Magnetom TRIO” (Siemens Medical Solutions, Erlangen, Germany) was used for data acquisition. Signal reception was performed with an 8-channel phased-array head coil and radiofrequency (RF) transmission with a body coil.

Functions included in MatLab (MathWorks, Natick, MA, United States), the FMRIB-Software-Library version 5.0.7 (FSL, Oxford) (Smith et al., 2004) and FreeSurfer version 6.0.1 (Athinoula A. Martinos Center for Biomedical Imaging, Boston) (Fischl et al., 2004) were used for analysis.

For B0 mapping, two gradient echo (GE)-datasets with different TE were acquired and processed with FSL PRELUDE and FUGUE: TE [1,2] = [4.89 ms,7.35 ms], TR = 560 ms, bandwidth = 200 Hz/pixel, field of view (FoV): 256 × 224 mm^2^, matrix size: 64 × 56, isotropic resolution = 4 mm, 40 sagittal slices (thickness: 4 mm, no gap), α = 60°, duration: 1:03 min.

B1-mapping was performed as reported in the literature (Volz et al., 2010). In summary, two GE-datasets were recorded (reference and magnetization prepared). The magnetization preparation consisted in an RF-pulse rotating the longitudinal magnetization by an angle β (nominal value: β_0_ = 45°), followed by a gradient spoiler. Thus, comparison of this dataset with the reference-data allows for the determination of the local β and B1 follows from deviations of β from β_0_. The other parameters were: TE = 5 ms, TR = 11 ms, α = 11°, bandwidth = 260 Hz/pixel, FoV, resolution and volume coverage as for B0-mapping, duration: 0:53 min.

For voxel-wise mapping of T1, the variable flip angle (VFA) method was used (Venkatesan et al., 1998), acquiring two spoiled GE-datasets at different excitation angles α_1,2_, resulting in different signal intensities (SI) in both datasets. The parameters were: TE = 6.7 ms, TR = 16.4 ms, α_1,2_ = [4°,24°], bandwidth = 222 Hz/pixel, FoV: 256 × 224 × 160 mm^3^, matrix size: 256 × 224 × 160, isotropic resolution = 1 mm, same volume coverage as for B0 and B1-mapping, duration (for both datasets): 9:48 min. Plotting of SI_i_/tan(α_i_) versus SI_i_/sin(α_i_) allowed for the calculation of T1 (Venkatesan et al., 1998). The respective preliminary T1-maps were corrected for inhomogeneities of B0 and B1, and for insufficient spoiling of the transverse magnetization (Preibisch and Deichmann, 2009).

T2-mapping was based on the acquisition of four T2-weighted fast spin echo datasets with different TE: TE = [13,67,93,106] ms, TR = 10 s, refocusing angle = 160°, bandwidth = 176 Hz/pixel, FoV: 256 × 176 mm^2^, matrix size: 256 × 176, 69 axial slices (thickness = 2 mm, no gap), spatial resolution = 1 × 1 × 2 mm^3^, turbo factor: 13, duration per dataset: 1:32 min. Each dataset was acquired twice for subsequent averaging, yielding a total duration of 12:16 min. T2 was determined by exponential fitting of the TE-dependence of signal intensities in the four averaged datasets and corrected for the influence of stimulated echoes as described in the literature (Nöth et al., 2017).

For PD-mapping, a method described in the literature (Volz et al., 2012a) was used. To this aim, the PD-weighted datasets resulting from the VFA-acquisition with the lower excitation angle were corrected for T1-, T2^∗^- and B1-effects and for inhomogeneities of the receive-coil profile (RCP). For the correction of signal-losses in the VFA-data induced by T2^∗^-relaxation effects during the finite TE of 6.7 ms, two GE-datasets with different TE were acquired: TE [1,2] = [4.3 ms,11 ms], TR = 1336 ms, α = 50°, bandwidth = 292 Hz/pixel, FoV: 256 × 224 mm^2^, matrix-size: 128 × 112, 80 sagittal slices (thickness: 2 mm, no gap), same volume coverage as for B0-, B1-, and T1-mapping, isotropic resolution = 2 mm, duration for both datasets: 5 min.

Synthetic T1-weighted magnetization-prepared rapid gradient-echo (MP-RAGE) anatomies were obtained as described previously (Gracien et al., 2019), using B0-corrected T1-maps and pseudo-PD-maps derived from T1-data via the Fatouros equation (Fatouros et al., 1991; Volz et al., 2012b). The virtual acquisition-parameters assumed for the synthetic data were: TR = 1900 ms, TI = 900 ms, α = 9°, echo-spacing = 8.1 ms. All geometrical parameters (FoV, matrix size, spatial resolution, volume coverage) were identical to the respective parameters of the underlying T1-maps.

Additional conventional MRI-acquisitions comprised MP-RAGE (Mugler and Brookeman, 1990) and FLAIR-datasets obtained with the following parameters:

MP-RAGE: TE = 3.04 ms, TR = 1900 ms, TI = 900 ms, α = 9°, bandwidth = 170 Hz/pixel, FoV: 256 × 256 × 192 mm^3^, matrix-size: 256 × 256 × 192, isotropic resolution = 1 mm, duration 4:28 min.

FLAIR: TE = 353 ms, TR = 5000 ms, TI = 1800 ms, bandwidth = 930 Hz/pixel, FoV: 256 × 220 × 160 mm^3^, matrix size: 256 × 220 × 160, isotropic resolution = 1 mm, duration 7:12 min.

All datasets were inspected by a senior neuroradiologist and by an experienced neurologist to assure absence of artifacts, e.g., related to subject movement.

### Method for Improved FCD-Detection

Segmentation of the cerebral cortex and WM, identification of the boundary between WM and the cortex and measurement of the cortical thickness were conducted by applying the Freesurfer script “recon-all” to the synthetic MP-RAGE-data.

Since a previous study has shown that data smoothing facilitates FCD-detection (House et al., 2013), the method for FCD-detection described below was performed twice, using either the original qMRI maps or smoothed versions of these maps as input data. For each map (T1, PD or T2), smoothing was performed separately for WM- and non-WM-voxels with a subsequent combination of the smoothed subparts. To this aim, the WM-masks computed via Freesurfer and the corresponding non-WM-masks, obtained by logical negation of the WM-masks, were applied to the qMRI-maps for each subject to isolate WM and non-WM voxels. To further improve the non-WM T1- and PD-maps and the respective masks, voxels with T1-values above 2000 ms were excluded, since GM T1-values range from 1200 to 1600 ms (Volz et al., 2012a). This step reduces partial volume effects with CSF. For the same reason, voxels with T2-values above 300 ms (Gracien et al., 2016) were eliminated from the non-WM T2-maps and masks. To avoid edge errors by including zero voxels in the smoothing process, an edge preserving algorithm was used: both the respective qMRI-map (WM or non-WM) and its corresponding mask were smoothed separately (kernel with full width at half maximum of 1.5 mm), calculating subsequently the quotient (smoothed map divided by smoothed mask). Voxels outside of the respective masks were excluded. Finally, voxels of the WM- and non-WM maps were recombined.

The following algorithm was applied twice with different input data, using either the original qMRI-maps or smoothed versions of these maps:

After boundary-based coregistration of the T2-maps to the synthetic MP-RAGE-anatomies with BBRegister, original or smoothed versions of the qMRI-maps were used to obtain values of the three investigated parameters (T1, T2, PD) at four different positions:

(i) Inside the cortex, avoiding areas close to the inner and outer cortex boundary. For this purpose, the cortex was subdivided into layers which were labeled according to their respective positions inside the cortex, given in percent of the cortical thickness (0% corresponding to the WM/cortex-boundary and 100% to the outer surface of the cortex). This subdivision was performed with a resolution of 1%. Only qMRI values from layers between the 20% and the 40% mark were read and averaged.
(ii) Inside the cortex, from layers between the 60 and 80% mark, as described above.
(iii, iv) At the corresponding positions in WM, mirroring the cortex at the WM/cortex-boundary.

Standard deviations (SD) of these four values were calculated for each qMRI-parameter, each cortical vertex, and each subject and were saved in surface-datasets. Furthermore, the cortical thickness (T) was obtained vertex-wise by applying Freesurfer to the synthetic MP-RAGE-anatomies. The SD-values and T were then combined according to the following formula in a surface-based analysis:

$$Q=\frac{S\,D\,\left(T\,1\right)\times S\,D\,\left(T\,2\right)\times S\,D\,\left(P\,D\right)}{T}$$

**FIGURE 1:**
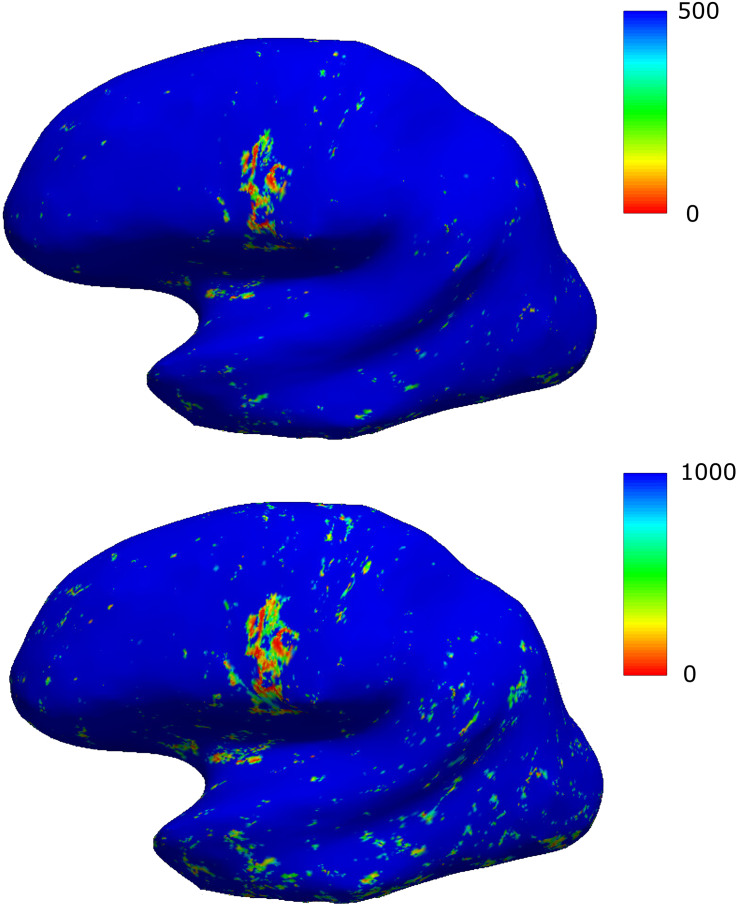
Results of the surface-based analysis demonstrated for a representative patient. Two different scalings are presented, the scaling in the first row resulting in an improved signal-to-noise-ratio. The area with focally decreased values (hot colors) corresponds to the location of an FCD.

A representative surface-based Q-map is demonstrated in Figure 1. The low values (hot colors) above the lateral sulcus corresponded to the location of an FCD. Figure 1 shows two different ranges for Q: 0–500 (top) and 0–1000 (bottom), the latter resulting in increased noise. Visual inspection revealed that FCD-areas are characterized by low *Q*-values. Thus, these datasets would already be suitable for visual FCD-detection. Still, as the Q-maps do not show anatomical information, they were rather used to enhance the signal in conventional FLAIR-datasets as described in the following paragraph.

To avoid zero-values for the subsequent division step, Q-values were increased by a minimal constant value of 0.0001. The surface-datasets were then projected into 3D-space with mri_surf2vol. To reduce effects of values above a threshold Q_0_ = 500, which had been empirically chosen (cf. Figure 1), the datasets were filtered by calculating the quotient Q_0_/Q, resulting in high or low values for Q < Q_0_ or Q > Q_0_, respectively. Very high values of the resulting quotient-maps above 1000 (corresponding to very low *Q*-values) were excluded to reduce artifacts in regions where cortical values cannot be read, such as areas of the medial hemispheres (corpus callosum and the third ventricle). Datasets were smoothed with a Gaussian kernel (sigma: 3 mm) and a constant value of 1.0 was added, resulting in the parameter R. The parameter R can be assumed to be approximately 1.0 in normal tissue and to be increased in FCD-areas (where Q is low). Thus, R is a suitable parameter for enhancing signal intensities in the clinical FLAIR-images.

The R-map was obtained twice, either with (R_*s*_) or without (R_*u*_) initial smoothing. The average of both R-maps was then multiplied with the conventional FLAIR-anatomy, which had previously been coregistered to the synthetic MP-RAGE-dataset, yielding signal enhancement in FCD-areas.

For an analysis of signal intensities, ROIs with the dimensions 2 × 2 × 1 mmł were manually chosen in the conventional FLAIR-datasets, representing regions where the FCDs are located and the corresponding contralateral cerebral control areas. ROIs were placed by an experienced neurologist and by a senior specialist in neuroradiology deciding by consensus. In these ROIs, mean values of signal intensities were read from the conventional and enhanced FLAIR-datasets, averaged across the group and compared via Wilcoxon tests. *P*-values below 0.05 were considered significant for all tests.

## Results

**FIGURE 2:**
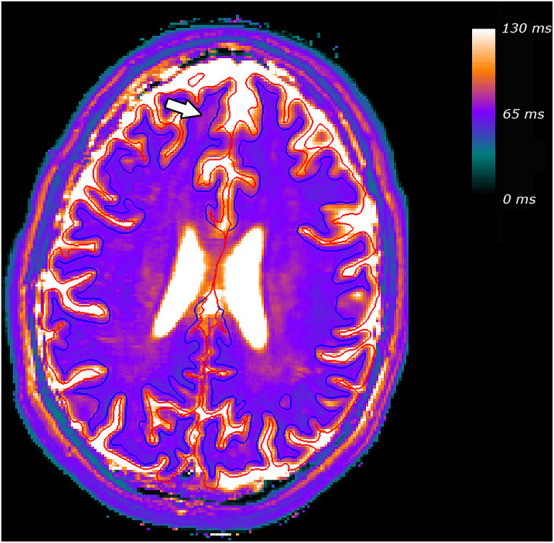
Tissue segmentation for a representative patient, superimposed on the T2 map. The blue line indicates the junction between cortex and WM and the red line the cortical surface. The FCD and the resulting increased subcortical T2-values are marked with an arrow.

To visualize the effect of FCD (marked with an arrow) on quantitative parameter values at the WM/cortex-junction, Figure 2 shows, for a representative patient, the result of the tissue segmentation superimposed on the T2-map. The blue line indicates the junction between cortex and WM and the red line the cortical surface. At the localization of the FCD, subcortical T2-values are increased, as a result of a smooth WM/cortex-junction.

For the group of patients with epilepsy, signal intensities across the FCD-ROIs were higher in the enhanced FLAIR-datasets (mean ± standard error of the mean: 202.41 ± 45.90) than in the conventional FLAIR-datasets (77.38 ± 6.16, *p* = 0.005) and higher than in the corresponding contralateral regions in the enhanced FLAIR-data (55.22 ± 2.35, *p* = 0.005). The FCD-signal in the enhanced in comparison to the conventional datasets was increased in 9/10 patients (relative increase: 66.27 ± 16.19%, range 22.53–146.90%), while no relevant increase could be observed for one patient (0.80%).

**FIGURE 3:**
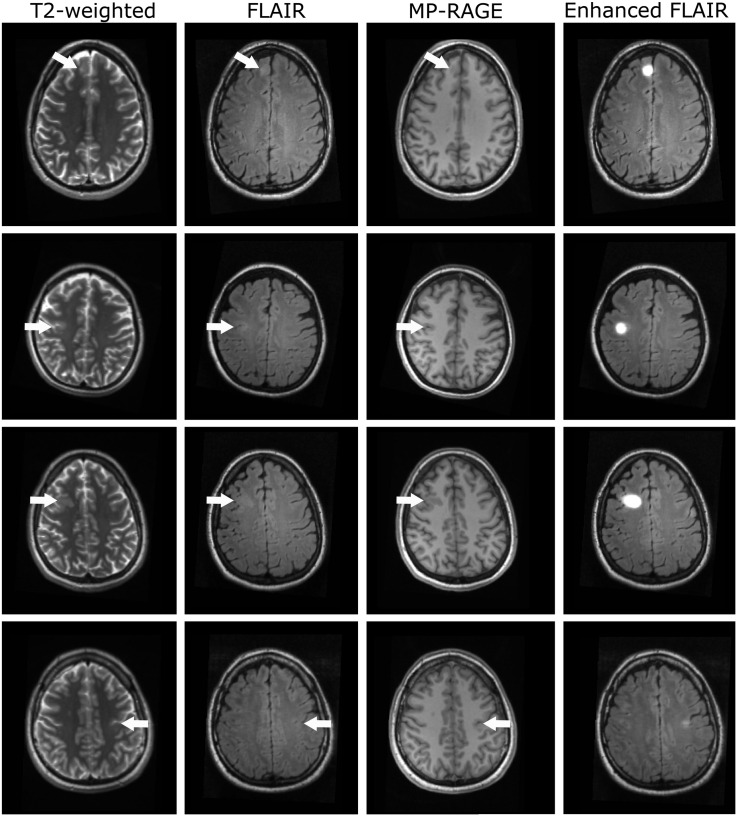
Representative datasets of four patients with FCD (rows). From left to right: T2-weighted, FLAIR-, MP-RAGE- and enhanced FLAIR-datasets. Arrows indicate the locations of the FCDs.

The final enhanced anatomies generated with this method for improved visualization of FCD and three clinical gold standard datasets (Wellmer et al., 2013) are presented in Figure 3 for four representative patients (rows), showing (from left to right) the conventional T2-weighted (TE = 67 ms), the FLAIR- and the MP-RAGE-datasets and the enhanced FLAIR-datasets. The subject in the first row corresponds to the subject shown in Figure 2. The FCD-areas are marked with arrows.

For the subjects shown in the first three rows, focal cortical (rows 1 and 2 in the conventional FLAIR-datasets, row 2 in the T2-weighted dataset) and subcortical (FLAIR/T2-weighted: rows 1 and 3) hyperintensities and cortical thickening (FLAIR/T2-weighted: row 2) were observed, indicative of FCD. Subcortical hypointensities (rows 1 and 3) and slight cortical thickening (rows 2 and 3) were observed in the conventional MP-RAGE datasets. For these three subjects, the signal intensity is strongly increased in the enhanced FLAIR-datasets in the FCD areas. The FCDs of the participants in the first and third row are clearly visible in the conventional MRI-datasets. In contrast, the lesion in the second row is less prominent. In this case, the strong signal in the enhanced dataset could help to guide the physician’s eyes when analyzing the images.

For the subject in the last row of Figure 3, diagnosed with an FCD in the left praecentral sulcus, only subtle cortical thickening was visible in the MP-RAGE dataset and a slight hyperintensity in the conventional FLAIR-image. However, such subtle changes might be easily missed when assessing the conventional clinical data. In contrast, the stronger signal in the synthetically enhanced FLAIR-dataset as demonstrated in the fourth column is indicative of this FCD.

The patients, whose data are shown in the first and second row, underwent surgical resection of the lesions after data acquisition and analysis. Histopathological assessment revealed FCDs type IIa (row 1) and type IIb (row 2).

**FIGURE 4:**
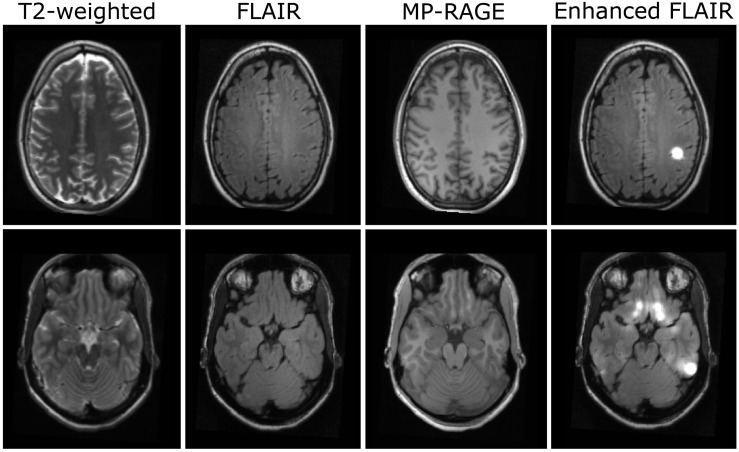
Examples for artifacts in datasets of two healthy subjects (rows). From left to right: T2-weighted, FLAIR-, MP- RAGE-, and enhanced FLAIR-datasets.

Some results, for which no correlate could be observed in the conventional clinical datasets, occurred both in patients and in the healthy control group. It is likely that most of these findings are false positive. Representative datasets of healthy subjects are presented in Figure 4: In particular, some false positive hyperintensities occurred in the enhanced FLAIR-images in regions where physiological properties such as closely spaced gyri result in a smooth WM/cortex-junction (Figure 4, first row). Furthermore, despite B0 correction of T1-data, residual B0-effects affecting T1-quantification and the segmentation in basal regions, resulted in some false positive results. A representative example is displayed in Figure 4 (second row).

## Discussion

The method presented in this preliminary technical study utilizes multiparametric qMRI-acquisition and surface-based analysis and combines assessment of the non-uniformity of qMRI-values across the junction between cortical GM and WM with vertex-wise measurements of the cortical thickness. This information is used to enhance the signal in conventional FLAIR-datasets in regions with a blurring at the WM/cortex-border or increased cortical thickness. We observed an increased signal in the enhanced FLAIR-datasets in regions where FCDs are located. Accordingly, the method might be helpful to visualize and detect FCD. Since the method was built utilizing the presented patients’ data, a clinical evaluation of the method based on this group of patients would not be appropriate and was beyond the scope of this study.

Importantly, qMRI-maps are intrinsically corrected for hardware effects such as inhomogeneities of the static magnetic field B0, the transmitted RF field B1 and the RCP (Cercignani et al., 2018). The respective hardware-effects in conventional datasets are problematic for FCD-detection because they yield signal non-uniformities which may impair tissue segmentation or the analysis of properties of the cortex and of the WM/cortex-border. In particular, pooled data acquired with different hardware may display different signal non-uniformities, thus rendering the analysis more difficult and requiring appropriate correction procedures. When using conventional MRI-data for improved FCD-visualization, such effects can be reduced with intensity correction/normalization-procedures, but a complete elimination is problematic. Accordingly, the use of qMRI-data which are free from such hardware effects should be particularly advantageous for FCD-detection.

A method using solely T1-data to derive maps of the cortical extent and of the smoothness at borders between WM and voxels with GM-characteristics was described recently (Nöth et al., 2020). In detail, T1-maps were used for a custom-built segmentation and creation of maps of the cortical extent. Furthermore, the T1-gradients at the WM/GM-border were calculated to generate maps for identification of regions with a blurring at this junction. The cortical extent and junction-maps were used to enhance the signal of synthetic DIR-datasets. While in the previous work the analysis was performed voxel-wise across the whole brain, the multiparametric method presented here is based on the reconstruction of the cortical and WM-surfaces. Another key difference is that the method presented here analyzes the junction between the cortex and the potentially abnormal WM, while the previous method creates a GM-characteristics-mask including GM and FCD-related abnormalities in WM and investigates the border between this mask and normal-appearing WM. Furthermore, junction- and thickness-analyses were combined in the present work to simplify the clinical assessment.

It should be noted that in the approach chosen here, the surface-datasets were first projected into 3D-space before smoothing was performed. A promising alternative approach which better respects the folded topology of the cortex (Lerch and Evans, 2005) would be to apply surface-based smoothing first. However, this approach is potentially problematic if an FCD is located on both sides of a sulcus. In this case, FCD-associated changes could be more closely spaced in 3D-space, forming a relatively compact area and thus high average *R*-values upon smoothing. In contrast, the FCD region might appear expanded in the surface-based dataset, which may reduce the effect of interest. Still, for further developments of the method, both approaches should be considered and tested.

In contrast to previous studies, the presented method enhances the signal of conventional FLAIR-datasets because clinicians are used to FLAIR-contrasts, which in general provide sufficient anatomical information for localization of the FCD. To pave the way of this method or other approaches toward the clinical application, future studies with larger cohorts will need to compare different methods to evaluate whether surface-based multimodal approaches are beneficial as compared to other techniques. These studies could also integrate diffusion tensor imaging (DTI) techniques to increase the sensitivity or to confirm the findings.

The method is not without limitations. As detailed in the results section, false positive findings may occur. Therefore, the enhanced datasets need to be compared carefully with conventional anatomies to confirm or reject each potential lesion. Furthermore, as the proposed method includes smoothing steps, the spatial extent of an FCD should not be estimated from the enhanced FLAIR-dataset, for example when planning surgical treatment. Since radiological evaluation of the presented method should not be based on the data used to develop the algorithm, future studies investigating different cohorts of FCD patients are required to evaluate the sensitivity and specificity of the method.

In summary, the presented multiparametric surface-based qMRI-method seems to be helpful to improve visualization of FCD. Accurate FCD-detection is of high relevance in the clinical routine because undetected lesions might in many cases result in wrong treatment decisions. Accordingly, the presented method might help to reduce false negative findings and improve the treatment of the respective FCD patients. Still, conventional anatomies remain the gold-standard for FCD-detection and the enhanced datasets should be carefully compared with routine datasets.

## Data Availability Statement

The datasets for this article are not available publicly or upon direct request because data sharing does not comply with the institutional ethics approval.

## Ethics Statement

The studies involving human participants were reviewed and approved by the Ethik-Kommission des Fachbereichs Medizin des Universitätsklinikums der Goethe-Universität. The patients/participants provided their written informed consent to participate in this study.

## Author Contributions

UN, SK, FR, RD, MW, and, R-MG contributed to the conception and design of the study. MM, RD, MW, and R-MG organized the study. MM, MW, and R-MG executed the study and acquired the data. RD calculated the quantitative maps. MM and R-MG designed the presented method and performed the statistical analysis. MM and R-MG wrote the first draft of the manuscript. All authors reviewed the statistical analysis and the manuscript, contributed to the manuscript revision and approved the submitted version.

## Conflict of Interest

EH has received speaker’s honoraria from BRACCO. SK has received speaker’s honoraria from Desitin and UCB and educational grants from AD-Tech, Bial, Brainlab, Desitin, Eisai, Epilog, GW, Siemens, Philipps, and UCB. FR has received honoraria for presentations and consultations from EISAI, UCB Pharma, Desitin Arzneimittel, Hexal, Novartis, Medtronic, GW-Pharma, Shire, Sandoz, and Cerbomed as well as research grants from UCB, European Union, Deutsche Forschungsgemeinschaft, European Science Foundation, and the Hessonian Ministries of Science and Arts and of Social Affairs and Integration. HS has received speaker’s honoraria from Bayer, Sanofi, and Boehringer Ingelheim. The remaining authors declare that the research was conducted in the absence of any commercial or financial relationships that could be construed as a potential conflict of interest.

## Abbreviations

CSF: cerebrospinal fluid
FCD: focal cortical dysplasia
FLAIR: fluid-attenuated inversion recovery
GE: gradient echo
GM: gray matter
ILAE: international league against epilepsy
MP-RAGE: magnetization-prepared rapid gradient-echo
PD: proton density
qMRI: quantitative MRI
RCP: receive coil profile
RF: radiofrequency
ROI: region of interest
SD: standard deviation
VFA: variable flip angle
WM: white matter.

## References

[B1] Ahmad R. Maiworm M. Nöth U. Seiler A. Hattingen E. Steinmetz H. (2020). Cortical changes in epilepsy patients with focal cortical dysplasia: new insights with T2 mapping. *J. Magn. Reson. Imaging* [Epub ahead of print]. 10.1002/jmri.27184 32383241

[B2] Cercignani M. Dowell N. G. Tofts P. (eds) (2018). *Quantitative MRI of the Brain: Principles of Physical Measurement.* Boca Raton FL: CRC Press.

[B3] Colombo N. Tassi L. Galli C. Citterio A. Lo Russo G. Scialfa G. (2003). Focal cortical dysplasias: MR imaging, histopathologic, and clinical correlations in surgically treated patients with epilepsy. *Am. J. Neuroradiol.* 24 724–733. 12695213 PMC8148665

[B4] Fatouros P. P. Marmarou A. Kraft K. A. Inao S. Schwarz F. P. (1991). In vivo brain water determination by T1 measurements: effect of total water content, hydration fraction, and field strength. *Magn. Reson. Med.* 17 402–413. 10.1002/mrm.1910170212 2062213

[B5] Fauser S. Schulze-Bonhage A. Honegger J. Carmona H. Huppertz H.-J. Pantazis G. (2004). Focal cortical dysplasias: surgical outcome in 67 patients in relation to histological subtypes and dual pathology. *Brain* 127 2406–2418. 10.1093/brain/awh277 15319274

[B6] Fischl B. Salat D. H. van der Kouwe A. J. Makris N. Ségonne F. Quinn B. T. (2004). Sequence-independent segmentation of magnetic resonance images. *Neuroimage* 23(Suppl. 1), S69–S84. 10.1016/j.neuroimage.2004.07.016 15501102

[B7] Gaitanis J. N. Donahue J. (2013). Focal cortical dysplasia. *Pediatr. Neurol.* 49 79–87. 10.1016/j.pediatrneurol.2012.12.024 23859852

[B8] Gracien R. M. Reitz S. C. Hof S. M. Fleischer V. Zimmermann H. Droby A. (2016). Assessment of cortical damage in early multiple sclerosis with quantitative T2 relaxometry. *NMR Biomed.* 29 444–450. 10.1002/nbm.3486 26820580

[B9] Gracien R.-M. van Wijnen A. Maiworm M. Petrov F. Merkel N. Paule E. (2019). Improved synthetic T1-weighted images for cerebral tissue segmentation in neurological diseases. *Magn. Reson. Imaging* 61 158–166. 10.1016/j.mri.2019.05.013 31075421

[B10] Hong S.-J. Bernhardt B. C. Schrader D. S. Bernasconi N. Bernasconi A. (2016). Whole-brain MRI phenotyping in dysplasia-related frontal lobe epilepsy. *Neurology* 86 643–650. 10.1212/WNL.0000000000002374 26764030 PMC4762421

[B11] Hong S.-J. Kim H. Schrader D. Bernasconi N. Bernhardt B. C. Bernasconi A. (2014). Automated detection of cortical dysplasia type II in MRI-negative epilepsy. *Neurology* 83 48–55. 10.1212/WNL.0000000000000543 24898923 PMC4114179

[B12] House P. M. Lanz M. Holst B. Martens T. Stodieck S. Huppertz H.-J. (2013). Comparison of morphometric analysis based on T1- and T2-weighted MRI data for visualization of focal cortical dysplasia. *Epilepsy Res.* 106 403–409. 10.1016/j.eplepsyres.2013.06.016 23891304

[B13] Huppertz H.-J. Grimm C. Fauser S. Kassubek J. Mader I. Hochmuth A. (2005). Enhanced visualization of blurred gray-white matter junctions in focal cortical dysplasia by voxel-based 3D MRI analysis. *Epilepsy Res.* 67 35–50. 10.1016/j.eplepsyres.2005.07.009 16171974

[B14] Kabat J. Król P. (2012). Focal cortical dysplasia – review. *Pol. J. Radiol.* 77 35–43. 10.12659/pjr.882968 PMC3403799 22844307

[B15] Kassubek J. Huppertz H.-J. Spreer J. Schulze-Bonhage A. (2002). Detection and localization of focal cortical dysplasia by voxel-based 3-D MRI analysis. *Epilepsia* 43 596–602. 10.1046/j.1528-1157.2002.41401.x 12060018

[B16] Lerch J. P. Evans A. C. (2005). Cortical thickness analysis examined through power analysis and a population simulation. *Neuroimage* 24 163–173. 10.1016/j.neuroimage.2004.07.045 15588607

[B17] Lerner J. T. Salamon N. Hauptman J. S. Velasco T. R. Hemb M. Wu J. Y. (2009). Assessment and surgical outcomes for mild type I and severe type II cortical dysplasia: a critical review and the UCLA experience. *Epilepsia* 50 1310–1335. 10.1111/j.1528-1167.2008.01998.x 19175385

[B18] Mugler J. P. Brookeman J. R. (1990). Three-dimensional magnetization-prepared rapid gradient-echo imaging (3D MP RAGE). *Magn. Reson. Med.* 15 152–157. 10.1002/mrm.1910150117 2374495

[B19] Nöth U. Gracien R.-M. Maiworm M. Reif P. S. Hattingen E. Knake S. (2020). Detection of cortical malformations using enhanced synthetic contrast images derived from quantitative T1 maps. *NMR Biomed.* 33:e4203. 10.1002/nbm.4203 31797463

[B20] Nöth U. Shrestha M. Schure J.-R. Deichmann R. (2017). Quantitative in vivo T2 mapping using fast spin echo techniques - A linear correction procedure. *Neuroimage* 157 476–485. 10.1016/j.neuroimage.2017.06.017 28602814

[B21] Preibisch C. Deichmann R. (2009). Influence of RF spoiling on the stability and accuracy of T1 mapping based on spoiled FLASH with varying flip angles. *Magn. Reson. Med.* 61 125–135. 10.1002/mrm.21776 19097220

[B22] Smith S. M. Jenkinson M. Woolrich M. W. Beckmann C. F. Behrens T. E. J. Johansen-Berg H. (2004). Advances in functional and structural MR image analysis and implementation as FSL. *Neuroimage* 23(Suppl. 1), 19. 10.1016/j.neuroimage.2004.07.051 15501092

[B23] Venkatesan R. Lin W. Haacke E. M. (1998). Accurate determination of spin-density andT1 in the presence of RF-field inhomogeneities and flip-angle miscalibration. *Magn. Reson. Med.* 40 592–602. 10.1002/mrm.1910400412 9771576

[B24] Volz S. Nöth U. Deichmann R. (2012a). Correction of systematic errors in quantitative proton density mapping. *Magn. Reson. Med.* 68 74–85. 10.1002/mrm.23206 22144171

[B25] Volz S. Nöth U. Jurcoane A. Ziemann U. Hattingen E. Deichmann R. (2012b). Quantitative proton density mapping: correcting the receiver sensitivity bias via pseudo proton densities. *Neuroimage* 63 540–552. 10.1016/j.neuroimage.2012.06.076 22796988

[B26] Volz S. Nöth U. Rotarska-Jagiela A. Deichmann R. (2010). A fast B1-mapping method for the correction and normalization of magnetization transfer ratio maps at 3 T. *Neuroimage* 49 3015–3026. 10.1016/j.neuroimage.2009.11.054 19948229

[B27] Wellmer J. Quesada C. M. Rothe L. Elger C. E. Bien C. G. Urbach H. (2013). Proposal for a magnetic resonance imaging protocol for the detection of epileptogenic lesions at early outpatient stages. *Epilepsia* 54 1977–1987. 10.1111/epi.12375 24117218

